# Whole-Exome Sequencing Identified Novel *CLMP* Mutations in a Family With Congenital Short Bowel Syndrome Presenting Differently in Two Probands

**DOI:** 10.3389/fgene.2020.574943

**Published:** 2020-12-15

**Authors:** Yao-Hung Chuang, Wen-Lang Fan, Yu-De Chu, Kung-Hao Liang, Yuan-Ming Yeh, Chien-Chang Chen, Cheng-Hsun Chiu, Ming-Wei Lai

**Affiliations:** ^1^Division of Pediatric Gastroenterology, Department of Pediatrics, Chang Gung Children’s Medical Center, Chang Gung Memorial Hospital, Taoyuan City, Taiwan; ^2^Genomic Medicine Research Core Laboratory, Chang Gung Memorial Hospital, Taoyuan City, Taiwan; ^3^Liver Research Center, Chang Gung Memorial Hospital, Taoyuan City, Taiwan; ^4^Department of Medical Research, Taipei Veterans General Hospital, Taipei, Taiwan; ^5^Institute of Food Safety and Health Risk Assessment, National Yang-Ming University, Taipei, Taiwan; ^6^Institute of Biomedical Informatics, National Yang-Ming University, Taipei, Taiwan; ^7^College of Medicine, Chang Gung University, Taoyuan City, Taiwan; ^8^Division of Pediatric Infectious Disease, Department of Pediatrics, Chang Gung Children’s Medical Center, Chang Gung Memorial Hospital, Taoyuan City, Taiwan

**Keywords:** *CLMP*, congenital short bowel syndrome, lactobezoar, non-sense mutation, long deletion mutation

## Abstract

Congenital short bowel syndrome (CSBS) is a rare condition characterized by an inborn shortening of bowel length with loss of intestinal functions, which often combines malrotation. CXADR-like membrane protein (*CLMP*) and filamin A (*FLNA*) gene mutations are the two major causes of this inherited defect. We presented two siblings with the older brother suffering from a laparotomy for bowel obstruction due to malrotation on the 17th day after birth. The younger sister encountered a laparotomy for lactobezoar at 6 months old. CSBS was diagnosed by measurement of the bowel length during the operations. Compound heterozygous *CLMP* mutations with the paternal allele harboring a long deletion across exon 3–5 and the maternal allele bearing a non-sense mutation of exon 3 (c.235C > T, p.Q79^∗^) were identified in both cases. They are the first reported familial CSBS caused by novel *CLMP* mutations in Taiwan.

## Introduction

Congenital short bowel syndrome (CSBS) is a rare condition described first in 1969 by Hamilton (Hamilton et al., 1969). The prevalence of CSBS is less than 1/1,000,000 (Orphanet.). Ischemic insult, defective neurenteric development, and myenteric plexus abnormalities could lead to such an intestinal defect (Tanner et al., 1976; Sansaricq et al., 1984). The diagnosis is often established at laparotomy exploration for intestinal obstruction in neonates or young infants because of non-specific presentations. Malnutrition and diarrhea are two other main features in CSBS resulted from the loss of small bowel length. Here we present two siblings with different manifestations: the older one presented with intestinal obstruction at age 16th day, whereas the younger one with intestinal obstruction caused by lactobezoar. CSBS was disclosed during operation in both. Whole-exome sequencing (WES) was performed for all, and whole-genome sequencing (WGS) was conducted for one member in the family to search for potential causative genetic defect(s) of CSBS.

## Materials and Methods

### Case Presentation

#### Case-1

A 4-day-old male neonate presented with jaundice. He was a product of a term pregnancy from non-consanguineous parents. His mother received regular antenatal examination, and no polyhydramnios was found. The pregnancy course was uneventful. He was born via a cesarean section with a birth bodyweight of 2.45 kilograms (kg).

**FIGURE 1:**
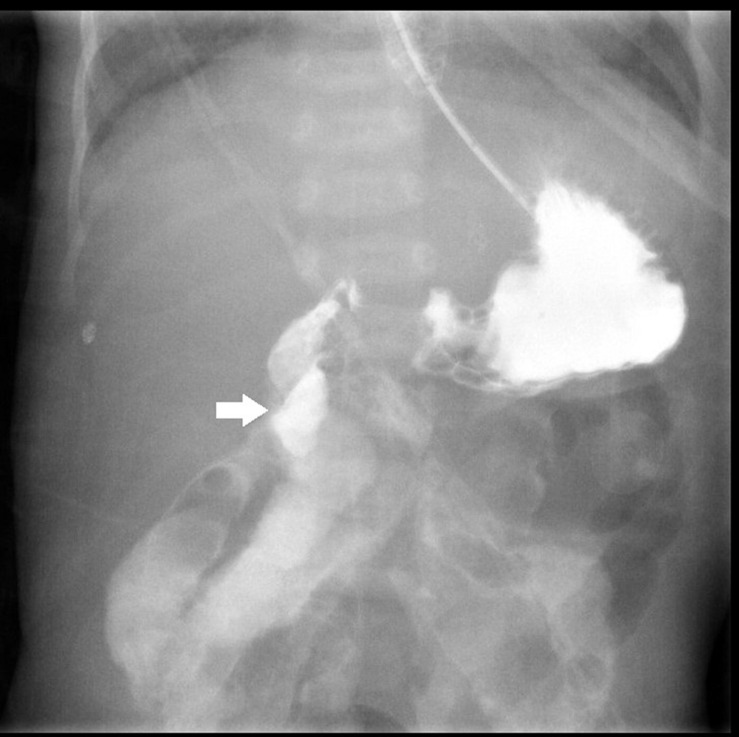
Upper gastrointestinal series with water-soluble contrast shows right-sided duodenojejunal junction with suspicious Ladd band compression at the 2nd portion of the duodenum (white arrow).

After birth, jaundice developed soon accompanied by frequent defecation, so that he was transferred to our hospital. Laboratory survey disclosed metabolic acidosis with hyperbilirubinemia (10.2 mg/dL) and hypernatremia (158 mEq/L). Prominent body weight loss from 2.45 to 1.99 kg (about 15.9%) was also noticed. Parenteral nutrition was administered since the hospitalization. After proper hydration, hyperbilirubinemia and electrolyte imbalance were corrected. However, he suffered from feeding intolerance. He had persisted watery diarrhea and developed bilious emesis on 16th days old. An upper gastrointestinal series revealed intestinal malrotation with suspicious Ladd band compression, as shown in Figure 1. A laparotomy with Ladd’s procedure was performed on the next day. An overall small bowel length of 30 cm and a colon length of 30 cm were measured during the operation. CSBS with malrotation was diagnosed.

After the operation, extensive hydrolysate formula containing medium-chain triglycerides was initiated 1 week later. The patient was discharged 65 days later. He grew steadily under home partial parenteral nutrition. The total duration of parenteral nutrition administration was 21 months. His body weight returned to an acceptable range at 2-year-1-month old (see Table 1).

**TABLE 1:** Clinical features in two siblings.

	**GA/BBW(g)**	**Presentation**	**Presenting age**	**Operation finding**	**SBL**	**Body weight recovery**
Case 1	Fullterm/2450	Jaundice watery diarrhea	4–16 days Old	Malrotation	30 cm	11.9 kg at 2 years1 month/old
		Bilious vomiting		Congenital short bowel		(15–50th percentile)
Case 2	Fullterm/2600	Non-bilious vomiting	6 months Old	Lactobezoar at terminal ileum	70 cm	8 kg at 12 months/old
		Constipation		Congenital short bowel		(15–50th percentile)

*GA, gestational age, BBW, birth body weight, SBL, small bowel length.*

#### Case-2

A 6-month-old infant, younger sister of case-1, was admitted to our hospital because of a sudden onset of projectile vomiting for 3 days. She was a full-term baby with a birth bodyweight of 2.6 kgs. The antenatal period, delivery, and post-natal period were uneventful. She was breastfed initially. However, frequent defecation was noted after birth. Thus, breastfeeding was substituted by hydrolyzed formula at 1 month old. Stool frequency decreased after that. Rice cereal was added into her formula at 5 months old.

**FIGURE 2:**
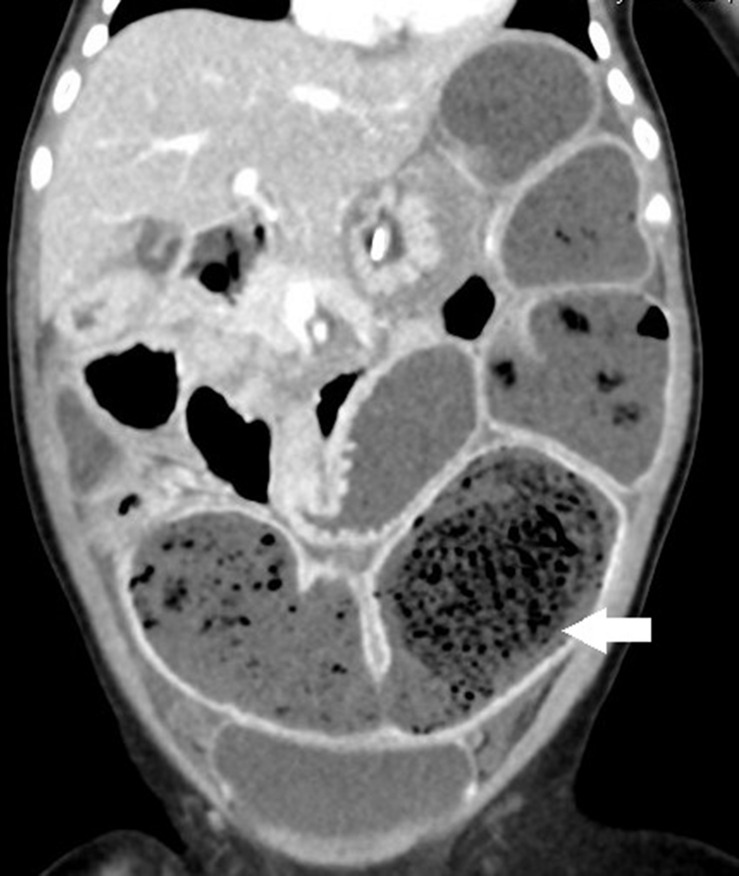
The coronal section of abdominal computed tomography (CT) discloses a prominent dilated bowel loop with air bubble-interspersed feces (lactobezoar) at the left lower quadrant (white arrow).

Failure to thrive was noticed at admission. An abdominal X-ray revealed prominent dilated small bowel gas. Nothing *per os* was ordered, and she was adequately hydrated with intravenous fluid. However, the appearance of emesis soon turned bilious. An abdominal computed tomography (CT) scan showed large bezoars in the dilated small bowel loops (Figure 2). We administered antibiotics, inserted a nasogastric tube, and tried rectal irrigation but in vain. A lower gastrointestinal series disclosed obstruction at the terminal ileum without a transitional zone. A laparotomy was performed on the 3rd day after admission, which revealed firm bezoar impacting the distal 10 cm of the ileum. Multiple intraoperative intestinal biopsies were performed and showed the presence of ganglion cells in all biopsies. The length of the small bowel was measured 70 cm. After the surgery, feeding was restarted 5 days later. Parenteral nutrition was administered for 27 days without subsequent home parenteral nutrition. She caught up with expected growth at 12 months old (see Table 1).

### Whole-Exome and Whole-Genome Sequencing

Genomic DNA, derived from blood samples after obtaining the parents’ consent, was extracted using the QIAamp DNA mini kit (QIAGEN, Germany, Cat: 51306) according to the manufacturer’s instruction. For both WES and WGS, 1 μg of genomic DNA was used to generate a DNA library with insert sizes around 150∼200 bp using SureSelect^*XT*^ Library Prep Kit (Agilent Technologies, United States, Cat: G9611A).

After in-solution enrichment of coding exons and flanking intronic sequences captured by the SureSelect Clinical Research Exome V2 kit (Agilent Technologies, United States, Cat: 5190-9491) and clustering of the index-coded samples using a cBot Cluster Generation System, or TruSeq PE Cluster Kit v4-cBot-HS (Illumina, United States, Cat: PE-401-4001) for WES and WGS, respectively, the libraries were then sequenced on the Illumina NovaSeq 6,000 platform with 150 bp paired-end sequencing. The cleaned sequence data were subsequently aligned and mapped to the reference genome (GRCh38) by Burrows-Wheeler aligner (BWA) using default options (Li and Durbin, 2009). GATK program was applied to perform base quality score recalibration, indel realignment, duplicate removal, mutation discovery, and genotype scoring using standard filtering parameters according to the GATK Best Practices recommendations.

Overall, in WES, in the four family members, 13.68G, 14.35G, 16.07G, and 15.79G bases were qualified and mapped to target exome regions with mean coverages of 112.3, 120.6, 135.6, and 133.9 times and thereby, respectively, identified 59307, 59673, 60241, and 60072 variants (McKenna et al., 2010). Common variants reported in dbSNP142 or the 1,000 Genomes Project with minor allele frequency (MAF) ≥0.001 were excluded. The Exome Aggregation Consortium (ExAC) database was used to confirm the novelty of variants. After removing common variants, candidate deleterious or pathogenic SNPs were identified using SIFT, PolyPhen2, LRT, MutationTaster, FATHMM, and M-CAP and CADD. The final variant classifications were based on the ACMG guidelines.

### Polymerase Chain Reaction and Sanger’s Sequencing

To confirm the c.235C > T substitution in CXADR-like membrane protein (*CLMP*) gene exon 3, PCR was performed using forward: 5′-CCACCGTGATGCATATGGCTA-3′ and reverse: 5′-CTACTTTGTGCCACTGGGAGT-3′ primers. To verify the deletion of *CLMP* gene exon 3–5, the PCR was conducted utilizing *CLMP*_del_F: 5′-AGGTC ACCCACCTGTCAAAG-3′ and *CLMP*_del_R: 5′-GGTGG GGGTTAGGAAAATGT-3′ primers. The amplified fragments were verified by Sanger’s sequencing.

## Results

**FIGURE 3:**
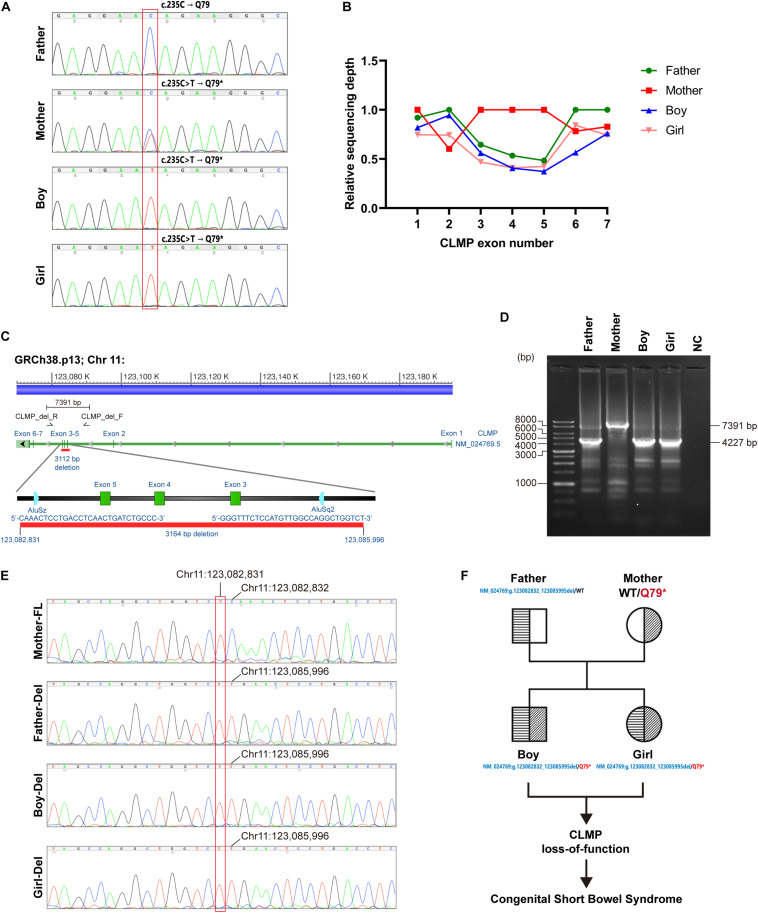
Identification of potential causative genetic mutation within the *CLMP* gene in our cases with CSBS. **(A)** Sanger’s sequencing confirmed the c.235C > T (p.Q79*) point mutation in exon 3 of *CLMP* open reading frame (ORF) in both siblings **(lower two panels)**; the father is wild type, and the mother is heterozygous. **(B)** The relative sequencing depth plot shows about one-half depths encompassing exon 3 to exon 5 of the father’s and two children’s reads than those of the mother’s reads. The average raw reads from individual bases in the same exon was normalized with the mean read of exon 1 from the mother to acquire the relative ratio. **(C)** The long deletion across exon 3–5 of the *CLMP* ORF was mapped by the girl’s whole-genome sequencing (WGS) (case-2). The primer set encompassing exon 3–5 for PCR is shown in the lower zoomed-in panel. **(D)** Gel electrophoresis of PCR products demonstrates large deletion (3164 bp) in the father and both siblings, compatible with the mapping by WGS. **(E)** Sanger’s sequencing confirmed the exon 3–5 deletion (chr11: 123,082,832_123,085,995del) in the father and two siblings. FL, full length; del, deletion. **(F)** The pedigree shows the inherence of *CLMP* genetic mutations in the family.

To complete genetic diagnosis and counseling for this family, we pursued W.E.S. for the affected siblings and their parents to identify any potential causative genetic mutation. After that, compound heterozygous mutations within the Coxsackie and adenovirus receptor (CXADR)-like membrane protein (*CLMP*) gene were called out. A single nucleotide substitution of c.235C > T (Chr.11: g.123084665 G > A) was identified in exon 3 of the *CLMP* open reading frame (ORF) in both children and their mother. Employing PCR followed by Sanger’s sequencing further confirmed this finding (Figure 3A). This mutation resulted in a premature stop codon (p.Q79^∗^). Annotating with gnomAD 3.0 and Taiwan Biobank revealed this pathogenic single nucleotide variant (SNV) is a novel mutation (Karczewski et al., 2020). A markedly reduced read-counts in exon 3–5 region in both children and the father highly implied a long deletion (Figure 3B). Verification using WGS of case-2 also supported this notion and further defined this long deletion’s exact flanking sequences (Figure 3C). Further validation using PCR followed by gel electrophoresis also agreed with this finding as the deletion product, with a size of 4227 bp, was only detected in samples from the father and both children but not from the mother’s, which generated a full-length (7391 bp) fragment (Figure 3D). The Sanger’s sequencing results directly showed the deletion occurred at Chr11:123,082,832-123,085,995 in amplicons from father and both children (Figure 3E). These results suggested a novel compound heterozygous NM_024769:g.123082832_123085995del/p.Q79^∗^ non-sens muta-tion in both probands and heterozygous genotypes in the parents, and it might thereby contribute to the development of CSBS (Figure 3F).

## Discussion

Congenital short bowel syndrome is a rare condition in infants, which implies a substantially functional loss of intestine, causing high morbidity and mortality. It is first described by Hamilton et al. in 1969, with two female siblings suffered from malrotation with CSBS in a non-consanguineous family (Hamilton et al., 1969). From 1969 to 2013, 46 cases with CSBS had been reported, and only 15 patients survived (van der Werf et al., 2015). Generally, the small bowel length in a term infant is about 190–280 cm (Reiquam et al., 1965; Siebert, 1980). In patients with CSBS, the mean small bowel length is approximately 57 cm (range 40–70 cm) (Schalamon et al., 1999). In CSBS, most patients were presented with vomiting, diarrhea, or failure to thrive. Malrotation is a common finding and usually results from the shortened small intestine or other developmental defects. Unlike acquired short bowel syndrome secondary to massive bowel resection, the diagnosis of CSBS usually is made after a laparotomy for intestinal obstruction or, in rare familial cases, by barium meal study with small bowel follow-through without surgery. From a systematic review of 61 CSBS cases, the survival rate has improved from 28.5 to 75% before and after 2008 because of advanced medical management (Negri et al., 2020). Both siblings in our report (with 30 cm and 70 cm small bowel length, respectively) survived with optimal nutritional recovery without the need for long-lasting parenteral nutrition (21 months and 27 days, respectively).

Lactobezoar (also called milk curd syndrome) is a rare condition first described by Wolf and Bruce in 1959 (Wolf and Bruce, 1959). Lactobezoar is mainly composed of undigested milk and mucus protein or fatty acid calcium stone (Heinz-Erian et al., 2012; Murase et al., 2013). It occurs mainly in neonates who are either premature or small for gestational age (Longardt et al., 2019). The case numbers decreased after the 1980s, possibly due to the advancement of neonatal care and the improvement of formula manufacturing (Iwamuro et al., 2015). The diagnosis of lactobezoar is based on radiographic findings (Towery and Chan, 2004). Lactobezoar is suspected of an imbalance between enteral food and digestive capacity (Hall and Ward, 2005). Immature intestine due to prematurity, overconcentrated formula, altered gastrointestinal physiology that affects gastric emptying and gastric acidity are the contributing factors causing milk curd syndrome (Schreiner et al., 1982; DuBose et al., 2001). To our knowledge, no association between the lactobezoar and CSBS has been reported. The possible cause of lactobezoar in case-2 could be due to concentrated cereal-added formula and imbalanced absorption/secretion of the short bowel.

Currently, the exact pathogenesis of CSBS remains unclear. Hamilton et al. (1969) hypothesized that the fetal intestinal development with elongation, rotation, and herniation was interrupted or delayed in patients with CSBS due to lack of space within the umbilical cord. Some reports speculated CSBS was the consequence of vascular accident or volvulus during the fetal stage (Sansaricq et al., 1984; Tiu et al., 1984; Kern et al., 1990). Later, *CLMP* and filamin A (*FLNA*) gene mutations are recognized as the two major disease-causing defects, either of which plays an important role in the intestine’s elongation. The *CLMP* gene locates on chromosome 11 (11q24. 1) and encodes a transmembrane protein (*CLMP*), which acts as an adhesion molecule. *CLMP* also co-localizes with tight-junction protein Zonula Occludens-1 and Occludin (Raschperger et al., 2004; Sze et al., 2008; Van Der Werf et al., 2012). To date, several *CLMP* mutations had been discovered including deletion in intron 1, deletion exon 2, c.28 + 1G > C, c.29-2A > G, c.589delA, c.371T > A (p.V124D), c.410G > A (p.C137Y), c.502C > T (p.R168^∗^), c.508C > T (p.R170^∗^), c.664C > T (p.R222^∗^), and c.1180G > A (Gharesouran et al., 2019; Negri et al., 2020). *CLMP* gene is expressed in the intestine in different stages during embryonic development. Van der Werf et al. demonstrated *CLMPa* (*CLMP* ortholog) knockdown in a zebrafish model resulted in a shorter length of body and intestine in the embryo, similar to the clinical phenotype of CSBS (Van Der Werf et al., 2012). Langhorst et al. (2018) had studied the *CLMP* function in a mouse model and demonstrated that *CLMP*-deficient mice presented malrotation instead of reduced intestinal length. The absence of *CLMP* also decreased expression of connexin 43 and connexin 45 in intestinal smooth muscle and connexin 43 in ureteral smooth muscle in the mouse model, which impaired calcium signaling of cell-cell communication and thus resulted in uncoordinated motility. Alves et al. (2016) also described 2 patients with *CLMP* mutations (c.508C > T; p. R170^∗^), who featured not only reduced bowel length but also ureteropelvic junction obstruction, possibly due to impaired ureteral peristalsis consistent with the mouse model. However, our report’s two siblings didn’t show any urinary tract anomaly in ultrasonography or CT scan.

Filamin A mutation is another genetic defect involved in the development of CSBS. *FLNA* encodes an actin-binding protein called *FLNA*, which functions in cell shape regulation, cell signaling, and migration (Alves et al., 2016). The genetic inheritance follows an X-linked recessive pattern. Compared to *CLMP* mutation, patients with *FLNA* mutations exhibit multiple organ anomalies in addition to a loss of intestinal function (Fox et al., 1998; Robertson et al., 2003; Kyndt et al., 2007; Bernstein et al., 2011). In contrast, in cases with *CLMP* mutation, the presentation is limited to the intestine.

Whole-exome sequencing of the whole family showed a compound heterozygous *CLMP* mutations in both probands (NM_024769:g.123082832_123085995del/c.235C > T, p.Q79^∗^ in exon 3) with the long-deletion segment inherited from the father, which was further supported by WGS followed by PCR and direct Sanger’s sequencing. Both mutations of the *CLMP* are novel in the genome aggregation consortium (GnomAD v3 and Taiwan Biobank) and are predicted to be pathogenic due to truncation and large deletion expected to cause defective or loss of protein function.

## Conclusion

In summary, our case-1, with a typical presentation of neonatal intestinal obstruction, was diagnosed as CSBS with malrotation after a laparotomy and established intestinal autonomy after 2-year home parenteral nutrition program. In contrast, his sibling, case-2, was confirmed with CSBS after laparotomy for an unusual presentation of lactobezoar-related intestinal obstruction at 6 months of age, which was never encountered in CSBS cases. Furthermore, we identified novel compound heterozygous *CLMP* NM_024769:g.123082832_123085995del/exon 3 non-sense mutation in both probands and heterozygous genotypes in the parents, albeit the pathogenesis of CSBS by these *CLMP* mutations needs further investigation.

## Data Availability Statement

The datasets for this article are not publicly available due to concerns regarding participant/patient anonymity. Requests to access the datasets should be directed to the corresponding author.

## Ethics Statement

Ethical review and approval was not required for the study on human participants in accordance with the local legislation and institutional requirements. Written informed consent to participate in this study was provided by the participants’ legal guardian/next of kin. Written informed consent was obtained from the individual(s), and minor(s)’ legal guardian/next of kin, for the publication of any potentially identifiable images or data included in this article.

## Author Contributions

Y-HC drafted the article. W-LF, Y-DC, K-HL, Y-MY, C-CC, and C-HC performed genetic and bioinformatic analyses. M-WL guided the entire essay and critically revised the manuscript. All authors analyzed the data and approved the final manuscript.

## Conflict of Interest

The authors declare that the research was conducted in the absence of any commercial or financial relationships that could be construed as a potential conflict of interest.

## Abbreviations

BWA: Burrows-Wheeler aligner
*CLMP*: CXADR-like membrane protein
CSBS: congenital short bowel syndrome
CT: computed tomography
DNA: deoxyribonucleic acid
*FLNA*: filamin A
GATK: Genome Analysis Tool Kit
GI: gastrointestinal
INDEL: insertions and deletion
SNV: single-nucleotide variation
WES: Whole-exome sequencing
WGS: Whole-genome sequencing.
